# Phosphorus nutrition of phosphorus-sensitive Australian native plants: threats to plant communities in a global biodiversity hotspot

**DOI:** 10.1093/conphys/cot010

**Published:** 2013-05-17

**Authors:** Hans Lambers, Idriss Ahmedi, Oliver Berkowitz, Chris Dunne, Patrick M. Finnegan, Giles E. St J. Hardy, Ricarda Jost, Etienne Laliberté, Stuart J. Pearse, François P. Teste

**Affiliations:** 1 School of Plant Biology, The University of Western Australia, 35 Stirling Highway, Crawley, WA 6009, Australia; 2 Centre for Phytophthora Science and Management, School of Biological Sciences and Biotechnology, Murdoch University, Murdoch, WA 6150, Australia; 3 Science Division, Department of Environment and Conservation, Locked Bag 104, Bentley Delivery Centre, WA 6983, Australia; 4 Astron Environmental Services, 129 Royal Street, East Perth, WA 6004, Australia

**Keywords:** Cluster roots, eutrophication, non-mycorrhizal plants, phosphite, *Phytophthora cinnamomi*, Proteaceae

## Abstract

South-western Australia harbours a global biodiversity hotspot on the world's most phosphorus (P)-impoverished soils. The greatest biodiversity occurs on the most severely nutrient-impoverished soils, where non-mycorrhizal species are a prominent component of the flora. Mycorrhizal species dominate where soils contain slightly more phosphorus. In addition to habitat loss and dryland salinity, a major threat to plant biodiversity in this region is eutrophication due to enrichment with P. Many plant species in the south-western Australian biodiversity hotspot are extremely sensitive to P, due to a low capability to down-regulate their phosphate-uptake capacity. Species from the most P-impoverished soils are also very poor competitors at higher P availability, giving way to more competitive species when soil P concentrations are increased. Sources of increased soil P concentrations include increased fire frequency, run-off from agricultural land, and urban activities. Another P source is the P-fertilizing effect of spraying natural environments on a landscape scale with phosphite to reduce the impacts of the introduced plant pathogen *Phytophthora cinnamomi*, which itself is a serious threat to biodiversity. We argue that alternatives to phosphite for *P. cinnamomi* management are needed urgently, and propose a strategy to work towards such alternatives, based on a sound understanding of the physiological and molecular mechanisms of the action of phosphite in plants that are susceptible to *P. cinnamomi*. The threats we describe for the south-western Australian biodiversity hotspot are likely to be very similar for other P-impoverished environments, including the fynbos in South Africa and the cerrado in Brazil.

## Abstract (precis)

South-western Australia harbours a biodiversity hotspot on severely phosphorus-impoverished soils. Threats include eutrophication due to phosphorus enrichment, due to increased fire frequency and spraying with phosphite to reduce the impacts of the introduced pathogen Phytophthora cinnamomi. We propose a strategy to work towards alternatives to phosphite for pathogen management.

## Introduction

**Figure 1:**
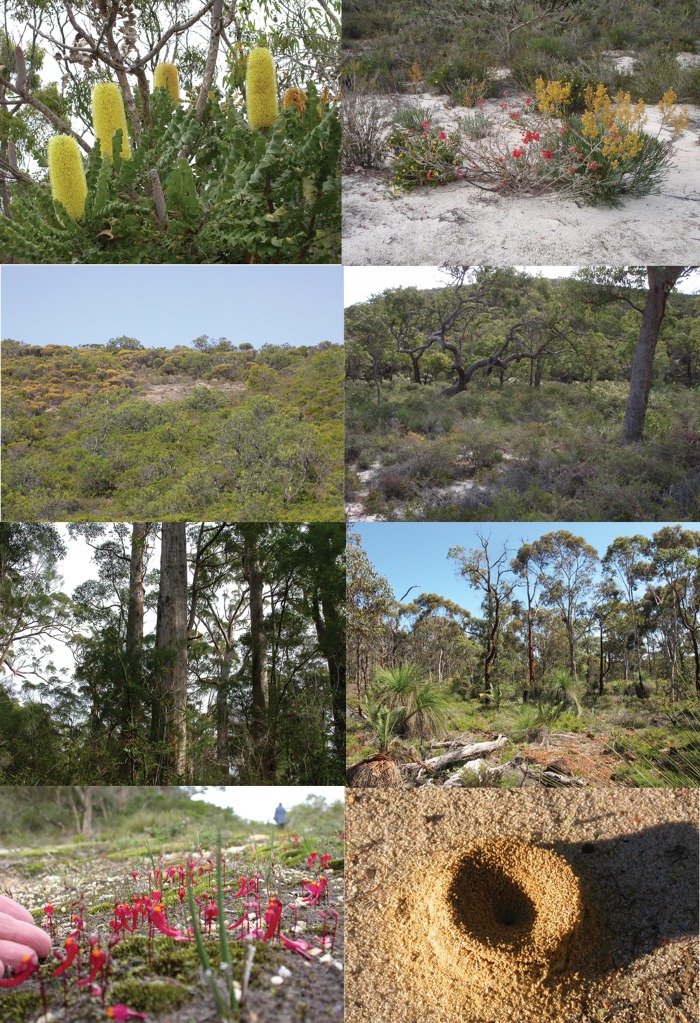
Plants and plant communities in south-western Australia's global biodiversity hotspot. The photographs are described from top left to bottom right. *Banksia grandis* (Proteaceae), an iconic species in banksia woodlands and jarrah forest which is very sensitive to elevated soil phosphate levels and susceptible to *Phytophthora cinnamomi*. The tree provides a rich source of nectar for birds, honey possums, and insects. A plant community on sandy soil in Lesueur National Park, showing red-flowering *Verticordia grandis* (Myrtaceae) and yellow-flowering *Stirlingia latifolia* (Proteaceae), amongst numerous other species. Low shrub on rocky substrate in Lesueur National Park. Woodland, with *Eucalyptus gomphocephala* (Myrtaceae) on sandy soil lower in the landscape, in Lesueur National Park. *Eucalyptus diversicolor* (Myrtaceae) in karri forest in the high-rainfall region of the far south-west in Western Australia. *Eucalyptus marginata* (Myrtaceae) in jarrah forest with a species-rich understorey. In seasonally wet habitats, numerous carnivorous species are found, including *Utricularia menziesii* (Lentibulariaceae). The greatest biodiversity is found on very sandy soil; the surface soil may not reveal the substrate underneath, but activities of ants provide information of the nature of the soil immediately below the surface. Photographs by Marion Cambridge and Hans Lambers.

South-western Australia harbours a global biodiversity hotspot, where exceptional numbers of endemic species are undergoing exceptional loss of habitat (Myers *et al.*, 2000; Myers, 2001; Hopper and Gioia, 2004). Due to the old age of the Australian continent, the relative stability of its geology, and the fact that most of Australia was last glaciated over 250 million years ago and has been above sea level for 145 million years (Miller *et al.*, 2005), many Australian soils are severely nutrient impoverished, and are especially low in phosphorus (P; Beadle, 1953). The most extremely P-impoverished soils occur in the region of the south-western biodiversity hotspot (Lambers *et al.*, 2010, 2012; Laliberté *et al.*, 2012; Fig. 1).

**Figure 2:**
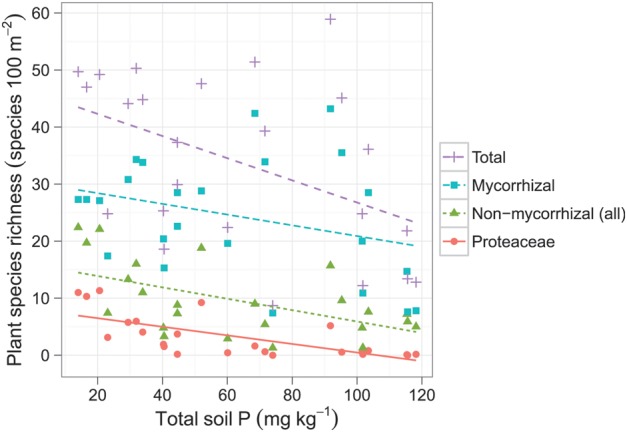
Plant diversity and soil phosphorus (P) status in south-western Australia's global biodiversity hotspot. Note the relative abundance of non-mycorrhizal species on soils with the lowest phosphorus content. Modified after Lambers *et al.* (2010).

In Western Australia's biodiversity hotspot, the greatest plant diversity is found on the most severely P-impoverished, sandy soils (Lamont *et al.*, 1977; Lambers *et al.*, 2010; Fig. 2). A similar pattern of increasing plant diversity with lower soil P status has been found for nutrient-poor habitats in Royal National Park, in New South Wales, in eastern Australia, one of the first sites in the world set aside to become a national park in 1879 (Adam *et al.*, 1989; Le Brocque and Buckney, 2003). This increase in plant diversity with lower soil fertility most probably reflects a global pattern (Huston, 1994; Grime, 2002; Wardle *et al.*, 2004; Laliberté *et al.*, 2013). For example, several long-term soil chronosequences worldwide show increases in local plant species richness with soil age, which is associated with lower soil [P] (Wardle *et al.*, 2008; Laliberté *et al.*, 2013). These patterns raise the possibility that P limitation *per se* might promote the coexistence of plant species (Olde Venterink, 2011). This may be related to the fact that P occurs in soil in many chemical forms and that several different P-acquisition strategies exist that can target specific forms (Lambers *et al.*, 2006, 2008).

One important P-acquisition strategy is the symbiotic association with mycorrhizal fungi, which can enhance a plant's ability to acquire P from low-P soil (Lambers *et al.*, 2008; Smith and Read, 2008). Although mycorrhizal plant species occur in P-impoverished soils from south-western Australia, non-mycorrhizal species are particularly successful on the most P-impoverished soils (Lambers *et al.*, 2006, 2010). This is explained by the ‘mining’ strategy of the non-mycorrhizal species, as opposed to a ‘scavenging’ strategy of mycorrhizal species (Lambers *et al.*, 2008), as detailed below. As a consequence, a significant proportion of south-western Australia's exceptionally rich plant biodiversity is represented by non-mycorrhizal species that show special adaptations to acquire P (Lambers *et al.*, 2010). Most of these species are found nowhere else on Earth (Hopper and Gioia, 2004), and therefore deserve special conservation attention (Hopper, 2009). In order best to conserve this important element of the south-western Australian flora, it is necessary to understand the P nutrition of these species in order to evaluate better the threats that they face. In this review, we first describe the adaptations exhibited by non-mycorrhizal species that allow them to grow successfully in P-impoverished soils. We then describe how many of these species show high sensitivity to P, leading to P toxicity. Finally, we discuss two major threats faced by these species, namely eutrophication through P fertilization, and the disease caused by the introduced soil-borne plant pathogen *Phytophthora cinnamomi* (Coates and Atkins, 2001), which is managed by large-scale application of phosphite, a more reduced form of P that can be oxidized to phosphate (Table 1).

**Table 1:** Two major threatening processes affect the biodiversity in the south-western Australian biodiversity hotspot and similar severely P-impoverished regions elsewhere in the world

Threatening process	Cause(s)	Physiological studies	Management options
Eutrophication due to increased soil phosphorus concentrations	Increased fire frequency	Revealing the cause of extreme P sensitivity of some Australian species: low capacity to down-regulate P uptake	Reduce frequency of controlled burning
		Competition among native species	Temporal monitoring of composition of the vegetation community
		Weed invasion as dependent on soil nutrient availability	Vegetation monitoring and eradication strategies in sensitive areas
	Fire suppressants	Effects on seed germination and persistence of P in soil	Replace P-containing suppressants by ones that do not contain P
	Storm water run-off from road verges and agricultural land	Seedling performance as dependent on P availability	Redirect storm water
	Dust from roads	Seedling performance as dependent on P availability	Use building material with minimal P content
	Application of phosphite to combat *P. cinnamomi*	Assess whether slow-growing P-efficient species are replaced by less P-efficient faster-growing ones	Replace P-containing fungicide (phosphite) by ones that do not contain P
*Phytophthora cinnamomi*	Introduction of an alien pathogen with a very large host range, particularly in the largely P-sensitive Proteaceae	Assess life cycle and performance of *P. cinnamomi*	Containment/eradication: (i) management of water flows to prevent water movement across the landscape from infested areas to disease-free areas; (ii) use of soil fumigants to treat spot infestations; and (iii) use of herbicides and total plant removal (fallow soils) for 2–3 years to starve the pathogen of living host tissues
	Spread of the pathogen by humans and feral animals	Mechanisms of phosphite action to boost the plant's immune system	Replace P-containing fungicide (phosphite) by ones that do not contain P
		Identification of most vulnerable plant communities; prioritization of research to those species and habitats	Use of measures to stop further spread of the pathogen

Several factors can lead to eutrophication, some of which are discussed in detail. Physiological studies allow a better understanding of plant responses to threatening processes. These should ultimately guide management options with conservation outcomes. The physiological studies that are listed are discussed in the main text.

## Adaptations to extremely ­phosphorus-impoverished soils

A plant family that is richly represented on severely P-impoverished soils in south-western Australia and similar landscapes in South Africa is the Proteaceae (Cowling and Lamont, 1998). Proteaceae species occur as major ecosystem components, often as keystone species. They are evergreen woody plants, ranging in size from prostrate shrubs to trees (Cavanagh and Pieroni, 2006; Collins *et al.*, 2008). After fires, Proteaceae species regenerate either by sprouting epicormic shoots or by releasing seed from woody follicles (Lamont and Markey, 1995). Proteaceae species contribute prominently to ecosystem functioning, such as fixation of carbon, provision of an architectural structure or habitat, and sharing and recycling of resources to minimize net nutrient loss, especially, from the system (Shearer *et al.*, 2009, 2012). Flowers provide food for birds, mammals, and insects (Hopper, 1980; Whelan and Burbidge, 1980; Ladd *et al.*, 1996; Cavanagh and Pieroni, 2006; Collins, 2008).

Most of the species in the Proteaceae family produce proteoid roots (Purnell, 1960) and almost all of them are non-mycorrhizal (Shane and Lambers, 2005a). An exception is the cluster-rooted mycorrhizal species *Hakea verrucosa*, which grows on ultramafic rocks that are rich in nickel (Boulet and Lambers, 2005). The release of carboxylic acids from its cluster roots would increase the solubility of nickel and harm the plants; in addition, mycorrhizal associations may protect plants to some degree against metal toxicity (e.g. Schutzendubel and Polle, 2002; Christophersen *et al.*, 2009). Proteoid roots are dense clusters of rootlets of determinate growth, which develop numerous root hairs (Lamont, 2003; Shane and Lambers, 2005a). Given that proteoid roots are not restricted to Proteaceae, the term ‘cluster roots’ is commonly used as an alternative (Shane and Lambers, 2005a). Cluster roots release vast amounts of carboxylates in an exudative burst (Watt and Evans, 1999; Shane *et al.*, 2004a); the carboxylates mobilize P that is sorbed onto soil particles, making it available for uptake by plant roots (Lambers *et al.*, 2006). Therefore, cluster roots effectively mine P that is unavailable for plants lacking this strategy. Outside the Proteaceae, cluster roots in Australia are found in Casuarinaceae (Reddell *et al.*, 1997), Fabaceae (Lamont, 1972), and Restionaceae (Lambers *et al.*, 2006). Cluster-root formation is suppressed in the presence of a high P supply (Watt and Evans, 1999; Shane and Lambers, 2005a), highlighting their important role for P uptake.

Dauciform (i.e. carrot-shaped) roots occur in some tribes of the non-mycorrhizal family Cyperaceae (Davies *et al.*, 1973; Lamont, 1974; Shane *et al.*, 2006b), another common family on nutrient-impoverished soils. Dauciform roots were probably first recognized for *Schoenus ferrugineus* (Cyperaceae) by Renner (1935), who thought they were a mycorrhizal structure (Pilzwurzel). Thereafter, they were described by Russian researchers (Selivanov and Utemova, 1969). Dauciform roots are morphologically very different from cluster roots, but functionally quite similar; they also release vast amounts of carboxylates in an exudative burst (Playsted *et al.*, 2006; Shane *et al.*, 2006a), and are suppressed in the presence of a high P supply (Shane *et al.*, 2006a).

Large amounts of carboxylates can also be exuded in the absence of specialized structures such as cluster roots and dauciform roots, e.g. in a range of *Kennedia* species (Fabaceae) from south-western Australia (Ryan *et al.*, 2012). These species without cluster roots differ vastly in the amount of carboxylates they release, but what they have in common is that their carboxylate exudation is reduced in the presence of arbuscular mycorrhizal fungi, which colonize the roots of some *Kennedia* species (Ryan *et al.*, 2012). This suggests a trade-off in plant carbon allocation for P acquisition to either mycorrhizal fungi or carboxylate release.

Manganese (Mn) accumulation is common in Proteaceae (Jaffré, 1979; Shane and Lambers, 2005b; Rabier *et al.*, 2007; Fernando *et al.*, 2010), as well as in Fabaceae with cluster roots, e.g. *Lupinus albus* (Gardner *et al.*, 1982) and *Aspalathus lineari*s (Morton, 1983). This is explained by the ability of their cluster roots to mobilize Mn (Gardner *et al.*, 1981; Grierson and Attiwill, 1989; Dinkelaker *et al.*, 1995). Leaf [Mn] might therefore provide an indication of the extent to which roots depend on carboxylate release to acquire P (Shane and Lambers, 2005b). Following this reasoning, we found that cluster-rooted Proteaceae and dauciform-rooted Cyperaceae along a 2 million year chronosequence (Laliberté *et al.*, 2012) have higher leaf [Mn] than their neighbours without any of the carboxylate-releasing strategies discussed above, regardless of soil age and P status (P. Hayes, E. Laliberté and H. Lambers, unpublished observations). We also found the same phenomena in non-mycorrhizal species that produce sand-binding roots. It is therefore likely that the functional significance of sand-binding roots (Shane *et al.*, 2011; Smith *et al.*, 2011) within an ecosystem is also, at least partly, that of mobilizing P through carboxylate release, but this requires further research.

The high abundance of Proteaceae in severely P-impoverished landscapes is not accounted for exclusively by their very efficient P-acquisition strategy. In addition, at least three other traits determine their P efficiency. First, their P-use efficiency in photosynthesis is amongst the highest ever recorded (Wright *et al.*, 2004; Denton *et al.*, 2007), which is partly accounted for by extensive replacement of phospholipids by lipids that do not contain P during leaf development (Lambers *et al.*, 2012). However, the decline in leaf P concentration during leaf development must also involve other metabolic changes, because the replacement of phospholipids is too small to explain the decrease entirely. Second, their long-lived leaves are very efficient and proficient at remobilizing P during leaf senescence (Denton *et al.*, 2007), which greatly increases the mean residence time of P within the plant. Third, their seeds, unlike their vegetative tissues, contain very high concentrations of P (Denton *et al.*, 2007; Groom and Lamont, 2010), allowing seedlings to grow without an external P source for a prolonged period (Milberg and Lamont, 1997).

## Phosphate sensitivity

Whilst Proteaceae and several other species that are endemic to south-western Australia and similar P-impoverished landscapes are very good at acquiring P and using it efficiently, many are extremely sensitive to P and readily display P-toxicity symptoms (Handreck, 1991; Parks *et al.*, 2000; Shane *et al.*, 2004b; Standish *et al.*, 2007; Hawkins *et al.*, 2008). Even a slight increase in the low P concentration that is common in their natural environment is enough to disturb their growth severely, and may cause death. However, whilst it is relatively common in P-impoverished landscapes, P sensitivity is by no means universal among species from P-impoverished habitats, and even some Proteaceae species are insensitive to elevated P supply, e.g. *Grevillea crithmifolia* (Shane and Lambers, 2006). The physiological mechanism accounting for P toxicity in higher plants is a low capacity to down-regulate their P uptake (Shane *et al.*, 2004c; Shane and Lambers, 2006; de Campos *et al.*, 2013). A low capacity to down-regulate P uptake is associated with a high capacity to remobilize P from senescing leaves, and *vice versa* (de Campos *et al.*, 2013). Whether this association is based on a mechanistic link involving the regulation of P transporters has yet to be explored.

Phosphorus enters the plant as inorganic phosphate (P_i_). Plants have both high-affinity and low-affinity mechanisms for the transport of P_i_ across the plasma membrane and into the cytosol against a steep electrochemical potential gradient (Cogliatti and Clarkson, 1983; Drew and Saker, 1984; Drew *et al.*, 1984). The high-affinity transport is facilitated by P_i_ transport proteins of the PHT1 family (Muchhal *et al.*, 1996). The proteins responsible for the low-affinity acquisition of P_i_ are unknown, but characterization mostly in heterologous systems suggests that they may also be PHT1 proteins (Harrison *et al.*, 2002; Rae *et al.*, 2003; Ai *et al.*, 2009; Preuss *et al.*, 2010). Caution, however, may be needed in drawing conclusions about P_i_ affinity determined in heterologous systems (Nussaume *et al.*, 2011). Once inside the cell, P_i_ is transported into various compartments by proteins of the PHT2, PHT3, and PHT4 families. It is widely accepted that P_i_ is moved through the plant body by a variety of PHT1 proteins. While all PHT1 proteins that have been examined are able to transport P_i_ into cells in normal physiological conditions, only some may be able to release P_i_ from cells, and then only in specific conditions, such as during senescence (Preuss *et al.*, 2010, 2011).

Every plant species that has been examined has multiple genes encoding high-affinity PHT1 proteins. *Arabidopsis* has nine *PHT1* genes, while there are 13 *PHT1* genes in rice. The exact number of *PHT1* genes in the P-sensitive Australian *Hakea prostrata* (Proteaceae) is unknown, but it is not atypical compared with these model plants (R. Jost, A. B. Siddique, B. Mirfakhraei, R. Pontré, H. Lambers and P.M. Finnegan, unpublished observations). Each *PHT1* gene has its own specific expression pattern (Karthikeyan *et al.*, 2002; Mudge *et al.*, 2002; Misson *et al.*, 2004; Xiao *et al.*, 2006; Morcuende *et al.*, 2007; Nussaume *et al.*, 2011). Most *PHT1* genes are strongly expressed in roots, but are also expressed in other cell types, tissues, and organs, with overlapping expression patterns among the members of the family (Nussaume *et al.*, 2011). The four *PHT1* genes of *H. prostrata* that have been characterized so far appear to follow these general expression trends (R. Pontré, B. Mirfakhraei, R. Jost, H. Lambers and P. M. Finnegan, unpublished observations).

A hallmark of the expression of nearly all *PHT1* genes is that their transcript abundance is repressed when tissues are exposed to high P_i_ and quickly derepressed when tissues are deprived of P_i_ (Karthikeyan *et al.*, 2002; Mudge *et al.*, 2002; Morcuende *et al.*, 2007; Nussaume *et al.*, 2011). This response to P_i_ availability provides an attractive mechanistic hypothesis for the low capacity of some native Australian plants to down-regulate their P uptake (Shane *et al.*, 2004c; Shane and Lambers, 2006; de Campos *et al.*, 2013). It may be that specific *PHT1* genes are not repressed by a sudden influx of P_i_ or that P_i_ exerts a positive effect on *PHT1* expression. Alternatively, PHT1 proteins present on the plasma membrane of specific cell types may not be removed from the membrane efficiently when P_i_ becomes more available. A putative repressor of PHT1 function, acting either directly or indirectly via PHO1, is the E2 ubiquitin conjugase PHO2 (Liu *et al.*, 2012). Remarkably, *pho2* mutants in *Arabidopsis thaliana* display a higher P_i_ uptake capacity than wild-type plants, leading to hyper-accumulation of P_i_ in leaves, resembling P-toxicity symptoms described for Proteaceae species (Dong *et al.*, 1998). This raises the possibility that plants adapted to very low P availability may not need this rather expensive module governing the down-regulation of P_i_ uptake. The finding that PHO2 is still expressed in *H. prostrata* (R. Jost, P. M. Finnegan and H. Lambers, unpublished observations) does not necessarily contradict this statement, because PHO2 seems to have functions other than the repression of PHO1 and P_i_ transport (as well as PHT1 transporters; Liu *et al.*, 2012). Further research is needed to understand the relative contribution of these processes to P_i_ toxicity in P-sensitive plants.

Each *PHT1* gene may have a specific function. In *Arabidopsis*, AtPHT1;1 and AtPHT1;4 are responsible for the bulk of P_i_ acquisition from the soil solution (Misson *et al.*, 2004; Shin *et al.*, 2004). Interestingly, *AtPHT1;4* transcript accumulation is not very responsive to changes in P_i_ availability (Woo *et al.*, 2012). This is also the case for rice *OsPHT1;1*, which is constitutively expressed in both root and shoot tissues. This transporter is responsible for the bulk of P_i_ uptake and translocation in rice during P-replete conditions and is still under the control of OsPHO2, but not OsPHR2 (Sun *et al.*, 2012). These findings indicate that there may be different regulatory pathways involved for inducible vs. bulk transport of P_i_. In Proteaceae species, a similar uncoupling of PHT1 expression from the P-starvation response mediated via the MYB transcription factor PHR1 could have taken place. Other transporters, such as AtPHT1;5, and perhaps the low-affinity barley HvPHT1;6, have an important role in the source-to-sink mobilization of P_i_ from senescing leaves (Huang *et al.*, 2011; Nagarajan *et al.*, 2011). Some *PHT1* genes are necessary for the rapid transport of P_i_ from the root to the shoot (H. Gaza, R. Jost and P. M. Finnegan, unpublished observations). In plants that form mycorrhizal associations, specific sets of *PHT1* genes are induced to transport P_i_ from the mycorrhizal partner to the plant (Smith and Barker, 2002; Javot *et al.*, 2007; Smith and Read, 2008; Pumplin and Harrison, 2009; Kobae and Hata, 2010; Loth-Pereda *et al.*, 2011). At the same time, other *PHT1* genes responsible for the acquisition of P_i_ from the bulk soil solution are repressed. This same response is observed during mycorrhization in the P-sensitive Australian *Eucalyptus marginata* (Myrtaceae; K. Kariman, R. Jost, S. J. Barker, P. M. Finnegan and M. Tibbett, unpublished ­observations).

## Species adapted to moderately ­phosphorus-impoverished soils

Most species in Australia's biodiversity hotspot have the capacity to establish a mycorrhizal symbiosis (Brundrett, 2009), but mycorrhizal species do not become dominant on the most severely P-impoverished soils (Lambers *et al.*, 2010). Their scavenging strategy allows them to acquire poorly mobile nutrients, such as P, that are at some distance from the root surface or in pores in the soil that are too small for root hairs to penetrate (Smith and Read, 2008), provided P is readily available and not strongly sorbed (Parfitt, 1979). Nevertheless, species that lack cluster roots or similar root specializations do co-occur with dominant Proteaceae (Lambers *et al.*, 2006, 2010), which do not form mycorrhizal associations (Shane and Lambers, 2005a). This may highlight the advantage for a plant to invest carbon to produce cluster roots, rather than investing carbon in an arbuscular mycorrhizal (AM) symbiosis in low-P soils. Furthermore, plants do not produce their roots in isolation, but rather interact with each other through them. However, the nature and outcome of this interaction for P uptake are unknown. The greater efficiencies in acquiring soil P by cluster-rooted Proteaceae could ultimately lead to a competitive exclusion of neighbouring mycorrhizal species. In addition, cluster-rooted Proteaceae may release a range of compounds that inhibit microbial activity and hence restrict mycorrhization, as found in *L. albus* (reviewed by Lambers *et al.*, 2013). However, as previously noted, total exclusion of mycorrhizal species does not occur on severely nutrient-impoverished soils, because many plant species co-occur without any obvious nutrient or growth deficiencies (Fig. 2).

While there are many possible explanations for why species of contrasting nutrient-acquisition strategies coexist in ancient, P-impoverished soils (Laliberté *et al.*, 2013), one of these is fitness equalization through resource sharing. For example, facilitation of P uptake has been demonstrated between agricultural cluster-rooted and AM species. Indeed, both P uptake and biomass are increased when wheat (*Triticum aestivum*) is intercropped with white lupin, due to the ability of the latter species to mobilize P with citric acid exuded by its cluster roots (Horst and Waschkies, 1987; Cu *et al.*, 2005). Phosphorus uptake from soil is also increased in AM wheat intercropped with chickpea (*Cicer arietinum*) growing on organic sources of P, and this is attributed to the release of acid phosphatases by chickpea (Li *et al.*, 2004). With regard to plant species coexistence in south-western Australian P-impoverished soils, this raises the following question: is there a potential facilitation of P_i_ uptake between cluster roots and the roots of a neighbouring AM plant, through the release of carboxylates, ribonucleases, and/or phosphatases? If so, then facilitation by plants that release carboxylates, ribonucleases, and/or phosphatases may slow down competitive exclusion of mycorrhizal species. We note that such fitness equalization would act as an ‘equalizing mechanism’ (*sensu* Chesson, 2000), because it would not, by itself, be sufficient to lead to stable coexistence. However, other stabilizing mechanisms that reduce inter-specific over intra-specific competition (*sensu* Chesson, 2000), such as partitioning of soil P between species of different nutrient-acquisition strategies (Turner, 2008; Lambers *et al.*, 2010), could also operate simultaneously and therefore lead to stable coexistence.

In pot experiments in a glasshouse study, Muler *et al.* (2013) found evidence for facilitation of the growth of ectomycorrhizal *Scholtzia involucrata* (Myrtaceae) by the cluster-rooted *Banksia attenuata* (Proteaceae). Interestingly, the positive effect of *B. attenuata* was not due to the mobilization of P, because addition of P did not enhance the growth of *S. involucrata*. Rather, the results showed facilitation of *S. involucrata* when grown with the cluster-rooted *B. attenuata*, due to enhanced acquisition of Mn and possibly other micronutrients. Leaf [Mn] of *S. involucrata* was increased in the presence of *B. attenuata*, and this increased [Mn] was associated with enhanced growth. It has yet to be explored how common the facilitation by carboxylate-releasing species is on severely P-impoverished soils. For example, facilitation does not appear to occur when Proteaceae plants have high levels of P in their tissue, possibly because this suppresses cluster-root activity. In a split-root experiment in a glasshouse, Wood (2011) found no evidence for facilitation of P uptake in the AM species *S. involucrata* or *Hibbertia subvaginata* in the presence of cluster roots of *B. attenuata*; neither biomass nor tissue [P] were positively related to the biomass of the neighbouring *B. attenuata* cluster roots. This result may have been due to the fact that the seedlings used by Wood (2011) had high initial P concentrations, especially in *B. attenuata*, with 1.2 ± 0.77 and 3.6 ± 0.77 mg g^−1^ dry weight in the young leaves and stems, respectively, i.e. 285% higher than plants growing in the wild (Lambers *et al.*, 2012).

What determines the fact that Proteaceae are more or less excluded from soils with higher P levels (Lambers *et al.*, 2010)? One possibility is that they might suffer P toxicity on such soils, as evidenced by pot trials with native species from P-impoverished locations on Hawkesbury Sandstone soils near Sydney, Australia (Thomson and Leishman, 2004). However, as not all Proteaceae are P sensitive (Shane and Lambers, 2006; Parks *et al.*, 2007), this is unlikely to be the full explanation. Proteaceae in south-western Australia's biodiversity hotspot tend to have a very high leaf mass per unit leaf area (Groom *et al.*, 1997; Lamont and Groom, 2002; Denton *et al.*, 2007; Lambers *et al.*, 2012), which tends to be associated with slow growth (Lambers and Poorter, 1992). Therefore, their inherently slow growth rate may lead to competitive exclusion of Proteaceae from less infertile sites. In the early 1950s, P fertilizers, as well as nitrogen and micronutrients, were added in various combinations to raise the fertility of an area of native heath vegetation in southern Australia. There was no significant response to the application of nitrogen or micronutrients, but a significant response to the combined application of nitrogen and P after 10 years. Fertilization with P showed major effects, which, collectively, gradually changed the heath vegetation, which initially had a significant component of Proteaceae species, towards an herbaceous sward over 22 years (Heddle and Specht, 1975). Below, we discuss how elevated soil P levels, for instance as a result of increased fire frequency, negatively impact most Proteaceae species.

## Eutrophication: role of phosphorus

Given that Proteaceae species rarely occupy habitats that are not severely P impoverished, it is envisaged that P enrichment has a negative impact on the performance of these species (Heddle and Specht, 1975). Indeed, urban storm water run-off has a negative impact on the growth and survival of species naturally occurring on P-impoverished Hawkesbury Sandstone soils, including *Banksia ericifolia* (Leishman *et al.*, 2004; Thomson and Leishman, 2004). Likewise, enrichment with P and other nutrients makes naturally P-impoverished habitats in urban bushland in Sydney, Australia, prone to invasion by exotic plants (King and Buckney, 2002).

In Bold Park, an area of urban bushland in Perth, Western Australia, increased fire frequency has led to elevated soil P levels and an abundance of exotic plants at the expense of species naturally occurring in P-impoverished soils, especially Proteaceae (Fisher *et al.*, 2006, 2009). A comparison of species composition in another urban bushland area in Perth, Kings Park, also exposed to increased fire frequency and the associated elevation in soil P status, between 1939 and 1999, shows a decline in four species of *Banksia* (Proteaceae) and an increase in the ectomycorrhizal trees *Eucalyptus gomphocephala*, *E. marginata* and *Corymbia calophylla* (Myrtaceae; Crosti *et al.*, 2007). Interestingly, there was an increase in the abundance of *Banksia* (formerly *Dryandra*) *sessilis*, showing that not all Proteaceae respond in the same manner to increased fire frequency. Similar changes have not been observed in pristine areas where fire frequencies have not increased.

One source of P in nutrient-poor areas is fire suppressants. Whilst not all contain P, those that do significantly enhance soil P, with an effect that persists for at least 12 months (Leach, 2013). In P-impoverished biodiverse areas, these P-containing fire suppressants should be avoided. Dust or run-off from roads, if they are constructed using nutrient-rich substrates, could also be a source of P, but this possibility has received very little attention.

In a study on the effects of water and nutrient incursion from agricultural land on adjoining native *Banksia* woodland in Western Australia, Grigg *et al.* (2000) found that numbers of species of native woody taxa and plant densities increased away from the agricultural area towards the pristine bushland. Total above- and below-ground biomass of woody species decreased away from the agricultural area, probably indicating a fertilization effect. Near the agricultural area, vegetation was appreciably depleted in leaf [Mn] compared with pristine bushland, suggesting that plants close to the boundary were relying less on carboxylate release for P acquisition. These data clearly show a negative impact of farming on nearby native bushland, with less P-sensitive species increasing in growth and more P-sensitive ones declining in abundance. Whilst the abundance of Proteaceae species in general was negatively impacted by incursion of nutrients and water from adjacent agricultural land, the abundance of *Banksia prionotes* increased (Grigg *et al.*, 2000). Interestingly, both *B. prionotes* and *B. sessilis*, which we discussed above, are among very few Proteaceae species that inhabit the young and comparatively fertile coastal dunes (Dixon, 2011) in south-western Australia, which are relatively nutrient rich in comparison to the much older and strongly weathered and acidic dunes on the Swan coastal plain and areas further inland (Laliberté *et al.*, 2012; Lambers *et al.*, 2012).

**Figure 3:**
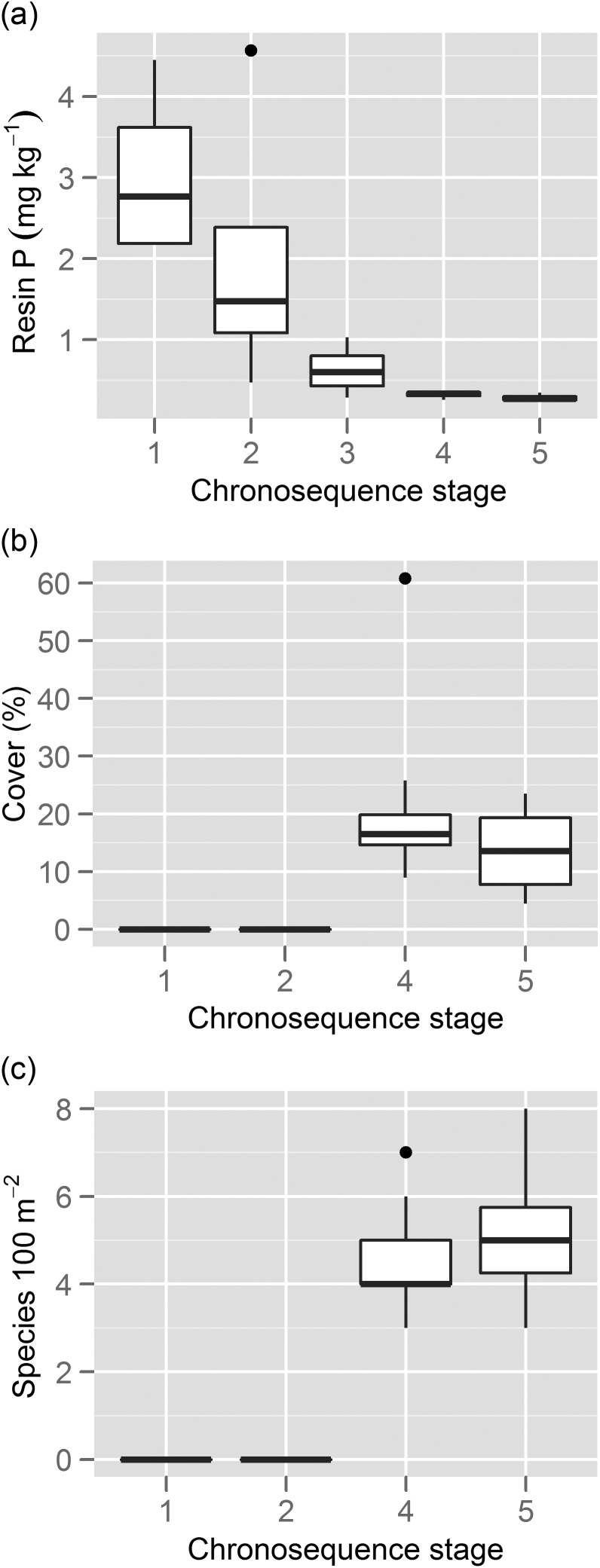
Changes in resin P (i.e. readily available P; **a**), percentage canopy cover of Proteaceae species (**b**), and species richness of Proteaceae (**c**) along five stages of the Jurien Bay 2 million year dune chronosequence in south-western Australia (Laliberté *et al.*, 2012). Data for (a) are from Laliberté *et al.* (2012), whereas data for (b) and (c) are from 50 10 m × 10 m plots (i.e. five chronosequence stages × 10 replicate plots) of a recent vegetation survey (G. Zemunik; unpublished data). Stages 1–3 represent Holocene (<7000 years) dunes, whereas stages 4–5 represent Pleistocene dunes (>120 000 years; Laliberté *et al.*, 2012).

The Jurien Bay dune chronosequence in south-western Australia provides a natural P-availability gradient that is driven by differences in soil age (Laliberté *et al.*, 2013). The sequence is formed of neighbouring coastal dune systems that were deposited at different periods during the last 2 million years (Laliberté *et al.*, 2012, 2013), along which changes in the relative abundance and diversity of Proteaceae species can be quantified. The dune chronosequence follows the classical model of long-term soil development (Walker and Syers, 1976), in that total soil P is relatively high in young soils, and decreases gradually to become the limiting nutrient in old soils (Laliberté *et al.*, 2012; Fig. 3a). Vegetation surveys along this dune chronosequence (G. Zemunik, unpublished data) clearly show that the relative abundance and species richness of Proteaceae species increase in older, more P-impoverished soils (Fig. 3b and c). Whether the lower relative abundance and diversity of Proteaceae species in younger dunes reflect their higher P availability or other soil conditions (e.g. high pH, high [calcium]) remains to be evaluated. What we can dismiss as a possible explanation for greater diversity on older dunes is that evolutionary processes continued for a longer period on the older dunes. Almost all of the species that occur on those older dunes also occur further inland, and they did not evolve locally on the older dunes themselves (Hopper and Gioia, 2004). Conversely, the habitat now provided by the younger dunes would be very similar to that of the older dunes when they were young (Laliberté *et al.*, 2013).

**Figure 4:**
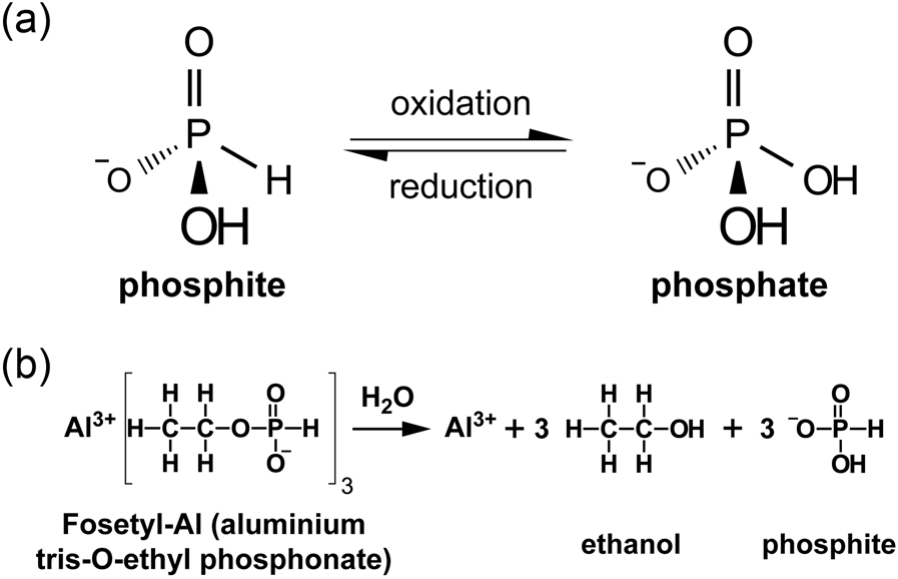
Phosphite, phosphate, and organic phosphonate. (**a**) Phosphite is less oxidized than phosphate and is not a source of phosphorus for plants. In soil, phosphite is microbially oxidized to phosphate, which then becomes available for uptake by plant roots. (**b**) Fungicides such as Fosetyl-Al are organic phosphonates (characterized by a stable C–P bond) that releases phosphite upon hydrolysis.

A special case of P enrichment of P-impoverished native bushland that arises as a result of spraying phosphite to reduce the impact of *P. cinnamomi* is discussed below. Phosphite is a more reduced form of P than phosphate (Fig. 4a); it is readily oxidized to phosphate by soil micro-organisms (White and Metcalf, 2007). Phosphite is an analogue of phosphate and is rapidly absorbed and translocated within the plant (Guest and Grant, 1991). It is mobile in the xylem and phloem (Ouimette and Coffey, 1989). However, in plants phosphite is not oxidized to phosphate, and there is no evidence that phosphite can be utilized by plants as a source of P (Guest and Grant, 1991; Förster *et al.*, 1998). Therefore, it is likely that phosphite concentrations will increase within the plant with regular applications of phosphite; this in turn will eventually be converted to phosphate in the soil once plants senesce or following fire. Regular spraying with phosphite, at rates of 24 kg ha^−1^, is similar to annual P-fertilization rates used by wheat growers in south-western Australia (Lambers *et al.*, 2010). Given that there is no export of P through cropping, applications of phosphite at these rates will inexorably enhance the total amount of P in the soil, leading to fertilization of plants. This situation will potentially have a negative impact on slow-growing species that do relatively better in P-impoverished habitats. That impact may not be due to their P sensitivity immediately, but may be through competitive interactions with faster-growing plants that lack strategies of extensive release of carboxylates and P-mobilizing enzymes (Heddle and Specht, 1975).

## *Phytophthora cinnamomi* as a major threat for Australia's biodiversity, and the use of phosphite to reduce the impact of plant disease

**Figure 5:**
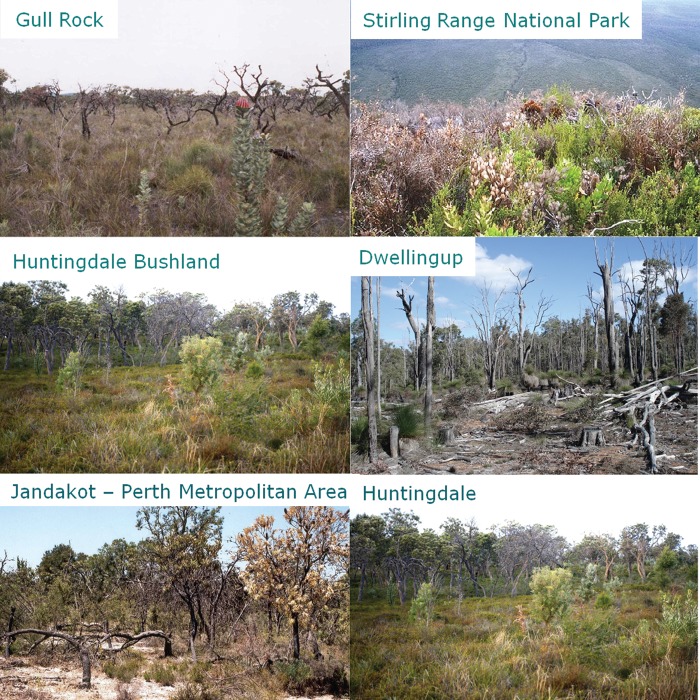
Devastating effects of *P. cinnamomi* occur throughout Western Australia's biodiversity hotspot, including national parks that exhibit the greatest biodiversity in the region and significant metropolitan bushland areas. Photographs by Chris Dunne, Department of Conservation, Western Australia.

The introduced plant-pathogenic oomycete *P. cinnamomi* (Stramenopila, Oomycota) is a major threat to biodiversity in Australia's global biodiversity hotspot (Coates and Atkins, 2001; Shearer and Fairman, 2007; Fig. 5), as well as elsewhere in Australia (Weste and Marks, 1987; Cahill *et al.*, 2008). It has been introduced into many of the global biodiversity hotspots, threatening susceptible rare flora and degrading plant communities, with severe consequences for fauna (Myers *et al.*, 2000). Shearer *et al.* (2004a) estimated that 40% (2284 species) of the flora of the South-West Botanical Province of Western Australia are susceptible, and 14% (800 species) are highly susceptible. The pathogen is listed as a ‘key threatening process to Australia's biodiversity’ by the Environment Protection and Biodiversity Conservation Act 1999 (Anonymous, 1999).

At this stage, phosphite is the only available chemical tool to combat the disease in natural systems in Australia (Hardy *et al.*, 2001; Shearer and Fairman, 2007). It has been marketed for several decades as the active ingredient released after hydrolysis of organic phosphonates (Fig. 4b; Cohen and Coffey, 1986) in fungicides such as Aliette^®^ (i.e. Fosetyl-Al, aluminium tris-*O*-ethyl phosphonate; BayerCrop Science) and effectively applied against plant diseases (downy mildews, and root and crown rots) in crops such as stone fruits, pineapples, and vegetables, as well as turf grasses and ornamentals. Phosphite can protect individual plants (Hardy *et al.*, 2001), but it does not stop disease progression in native vegetation (Shearer *et al.*, 2004b, 2006; Dunne *et al.*, 2010). Phosphite does reduce mortality due to *P. cinnamomi* (Pilbeam *et al.*, 2000; Wilkinson *et al.*, 2001; Barrett *et al.*, 2003), and it can slow the rate of disease centre extension and reduce the rate of plant mortality (Shearer *et al.*, 2004b). In the moderately resistant *Lambertia formosa* (Proteaceae) and the more susceptible *Lambertia inermis*, phosphite-induced protection is associated with increased superoxide release 8 h after inoculation, and increased phenylalanine ammonia lyase activity 24 h after inoculation (Suddaby *et al.*, 2008). In *E. marginata* (Myrtaceae), the effect of phosphite in controlling the pathogen is probably determined by the phosphite concentration at the host–pathogen interface. When phosphite concentrations within the roots are low, it primes the tissue for a stronger and faster defence response upon pathogen infection (Jackson *et al.*, 2000). Phosphite induces the host's defence against *P. cinnamomi* as well as a wide range of other pathogens, ranging from oomycetes to fungi and nematodes (Reuveni *et al.*, 2003; Aguín *et al.*, 2006; Oka *et al.*, 2007; Percival *et al.*, 2009; Lobato *et al.*, 2010). Phosphite-induced resistance can protect treated plants for extended periods of months or even years before re-application becomes necessary (Tynan *et al.*, 2001; Dunstan *et al.*, 2010). High phosphite concentrations in the root tissue act directly on the pathogen to inhibit its growth before it can establish an association with the host, and the host defences remain unchanged (Jackson *et al.*, 2000).

Although the term ‘fungicide’ is often used to describe the direct effect of phosphite on oomycete pathogens, it is in fact a biostat, because phosphite does not kill the pathogen, but only slows its growth. This is mainly explained by its impact on metabolism, most probably impairing phosphate homeostasis and metabolic reactions, at least partly through mimicking phosphate while not being metabolized (Griffith *et al.*, 1990; Barchietto *et al.*, 1992; Niere *et al.*, 1994). Despite the wide and successful use of phosphite to prevent the spread of *P. cinnamomi*, our understanding of the molecular mechanisms underlying the protective effect of induced resistance in the plant is scant. Studies in the model plant *Arabidopsis thaliana* have enabled the mode of phosphite action to be analysed at the molecular level. The plant hormone salicylic acid, a well-known signalling molecule in defence responses, and the transcription co-activator NPR1 (non-expressor of PR1), involved in the salicylic acid-dependent signalling pathway, are important components in the mechanisms leading to increased resistance after phosphite application. Mutant plants impaired in the accumulation of salicylic acid or its signalling cascade show reduced phosphite-induced resistance against the oomycete pathogen *Hyaloperonospora arabidopsidis* (Molina *et al.*, 1998; Friedrich *et al.*, 2001). Moreover, phosphite application can lead to a stronger and faster defence response in *A. thaliana* subsequently inoculated with *H. arabidopsidis* (Massoud *et al.*, 2012). This response involves the proteins EDS1 (enhanced disease susceptibility 1) and PAD4 (phytoalexin deficient 4), which are upstream of salicylic acid, and the mitogen activated protein kinase MPK4. This augmented activation of resistance mechanisms by pre-treatment with low levels of the inducing chemical, often leading to local and systemic immunity, has been known for a long time and termed ‘defence priming’ (Conrath *et al.*, 2006). Interestingly, this acquired primed state can be passed on to the offspring by epigenetic modifications such as DNA methylation (Luna *et al.*, 2012; Rasmann *et al.*, 2012; Slaughter *et al.*, 2012). This transgenerational induced resistance has the potential for an inheritable adaptation to pathogen pressure and might also explain the long-lasting effect of phosphite applications if similar epigenetic modifications are induced. Thus, understanding of the molecular mechanism of phosphite action has great potential. Current ‘omics technologies (i.e. genomics, proteomics, and metabolomics) allow determination of phosphite-induced changes in gene expression, and protein and metabolite abundances. Their application will allow a detailed description of the impact of phosphite on a whole-systems scale, and will help in the identification of key targets of phosphite, thus creating a knowledge base for the targeted development of alternative compounds. Although this type of approach is especially applicable to plant model species, such as *A. thaliana*, because of a highly developed genetic and molecular tool kit, similar approaches and translational knowledge transfer become increasingly applicable for non-model organisms (Lambers *et al.*, 2012).

As discussed above, phosphite is oxidized to phosphate by soil microbes, potentially shifting the balance between highly P-efficient, non-mycorrhizal species and less P-efficient ones. Finding an alternative to phosphite would prevent the indirect increase of P availability in native ecosystems. The need for such alternatives is also apparent from reports of oomycete pathogens developing resistance to phosphite, as has been observed for lettuce downy mildew (Brown *et al.*, 2004). Chemicals with similar defence-inducing properties, such as the salicylic acid analogue benzo-(1,2,3)-thiadiazole-7-carbothioic acid and the non-proteinogenic amino acid β-aminobutyric acid are available, and their efficacy has been studied (Conrath, 2009). So far, large-scale application, similar to phosphite, has not been successful, not even in agricultural systems that are easier to manage. Nevertheless, knowledge gained concerning these compounds will also improve our understanding of the mode of phosphite action and the identification of practical alternative compounds.

**Figure 6:**
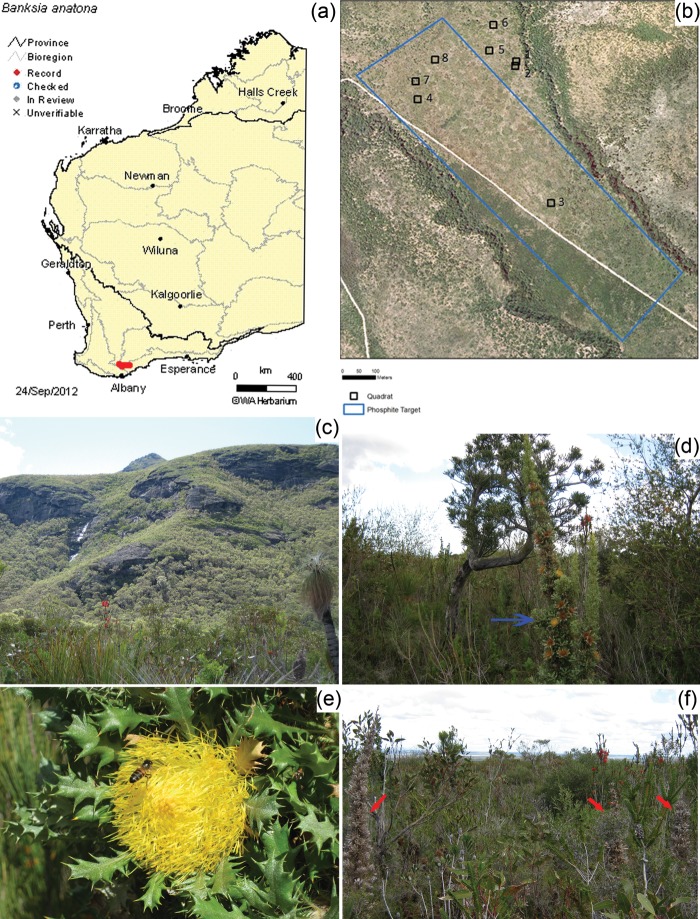
*Banksia anatona* (A.S.George) A.R.Mast & K.R.Thiele (cactus banksia) is an IUCN Red List Critically Endangered ranked species at extremely high risk of extinction in the wild, because of *P. cinnamomi* infestations. (**a**) The distribution of *B*. *anatona*, found only on the lower slopes of the Stirling Ranges in south-western Australia (DEC, 2012). (**b**) The location of monitoring quadrats inside and outside of an annually sprayed phosphite treatment target area. The target area contains a *P*. *cinnamomi* infestation. (**c**) View of the Stirling Range to the north of the target treatment area. (**d**) *Banksia anatona* (blue arrow) is an upright, non-lignotuberous shrub, growing up to 5 m tall, found growing on grey sand over gravelly shale and rocky silty clay loam (DEC, 2012). (**e**) Close-up view of *B*. *anatona* leaves and a flower. (**f**) *Banksia anatona* is extremely susceptible to *P. cinnamomi*. Red arrows indicate dead *B. anatona* as a result of *P. cinnamomi* infection. Text and image used with permission.

**Figure 7:**
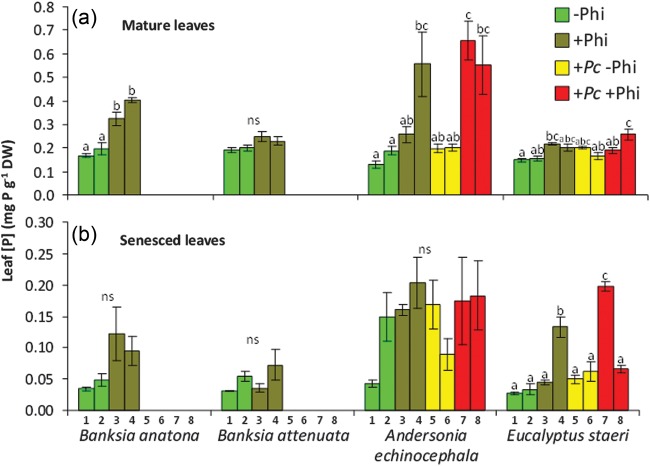
Leaf P concentration of mature (**a**) and senesced leaves (**b**) of *B. anatona*, *B*. *attenuata*, *Andersonia echinocephala*, and *Eucalyptus staeri* located on the lower slope of the Stirling Range, Western Australia; note the different scales for the two panels. The plants were located within quadrats (numbered 1–8; see Fig. 6b), growing naturally (–Phi), treated with phosphite (+Phi), infested with *P. cinnamomi* and not treated (+*Pc* –Phi), or infested and treated with phosphite (+*Pc* +Phi; *n* = 3). Error bars indicate SEM (*n* = 4); columns not sharing the same letter indicate significant differences between treatments within species according to Tukey's test (*P* = 0.05); ns, no significant difference. Different columns with the same colour refer to different quadrats (Fig. 6b) for the same treatment; three trees within each quadrat were sampled, and four leaves per plant were used. Missing bars are due to the fact that trees had succumbed to the pathogen before sampling had commenced.

The elevation of leaf [P] resulting from phosphite application has scarcely been investigated, but is evidenced in nature. In Stirling Range National Park, an IUCN Red List Critically Threatened species, *Banksia anatona* (A.S.George) A.R.Mast & K.R.Thiele, is at extremely high risk of extinction because of *P. cinnamomi* infestations. *Banksia anatona* exists ­naturally only on the lower slopes of the Stirling Range (Fig. 6a,c,d and e). Phosphite has been applied by aerial spraying to a target area for 15 years to slow the loss of a *B*. *anatona* community due to *P*. *cinnamomi* infestation (Fig. 6b and f). Samples taken from monitoring quadrats inside and outside the phosphite spray target area show that the [P] in mature and senesced leaves of *B*. *anatona* where *P*. *cinnamomi* is absent has significantly increased compared with unsprayed areas (Fig. 7a and b). Evidence of elevated leaf [P] can also be seen in other species, including *Banksia attenuata* R.Br, *Andersonia echinocephala* (Stschegl.) Druce and *Eucalyptus staeri* (Maiden) Kessell & C.A.Gardner. Samples were taken in 2011 from *B*. *anatona* and *B*. *attenuata* only in areas where *P*. *cinnamomi* was absent, because irrespective of whether or not phosphite had been applied, over the time since monitoring quadrats were established, *P*. *cinnamomi* had already killed all individuals in the infested quadrats. This example highlights the urgent need for alternative, practical and effective compounds that do not contain P to be developed to manage *P*. *cinnamomi* infestations to prevent both species loss and the elevation of the P status of ecosystems. Whilst the biomass of major plants in the community has increased following phosphite application, to date there is no firm evidence of a change in plant community composition, possibly because of the low productivity of the system and the relatively long time it takes to achieve such a shift in community structure.

With the exact molecular mode of phosphite action being unknown, it is important also to consider its impact on P-signalling networks in plants (Ticconi *et al.*, 2001; Varadarajan *et al.*, 2002). Disturbances in nutrient ­homeostasis have recently been shown to impact directly on ­­plant–pathogen interactions (Pannell and Ewing, 2006; Van Damme *et al.*, 2009; Stuttmann *et al.*, 2011; Álvarez *et al.*, 2012; Kruse *et al.*, 2012). Therefore, phosphite may interact with P_i_-signalling components that could trigger the suggested priming of defence responses. Phosphate-signalling networks can include both local and systemic signalling pathways, and it has been assumed that local responses are triggered upon the recognition of P_i_ itself, while systemic responses may be triggered by changes in hormone levels or in a yet to be identified organic phosphate ester pool (Liu *et al.*, 2010; Thibaud *et al.*, 2010; Chiou and Lin, 2011; Nagarajan and Smith, 2012). Given that phosphite cannot be metabolized by plants, it has been assumed only to interfere with the local sensing of P_i_ itself; therefore, it has long been considered an excellent tool to distinguish between different sensors (McDonald *et al.*, 2001; Ticconi *et al.*, 2004). Unfortunately, P_i_ is a strong inhibitor of methylphosphonate (an organic analogue of phosphite) transport with an apparent *K*_i_ of 5 µM (Pratt *et al.*, 2009). Phosphite and methylphosphonate also show different accumulation patterns in subcellular compartments compared with P_i_ and are influenced by the presence of P_i_ according to detailed nuclear magnetic resonance studies using cell culture systems (Danova-Alt *et al.*, 2008; Pratt *et al.*, 2009). These findings suggest that any observed effects of phosphite need to be interpreted with caution. They also suggest that the P status of the plant is critically important for phosphite acquisition and allocation, and may explain the varying degree of success of plant protection in the field. Starving plants of P_i_ before the application of phosphite to circumvent some of these issues has been considered by Varadarajan and co-workers (2002); however, the strong overall growth inhibitory effects of phosphite on P-starved plants (Thao and Yamakawa, 2009) make it necessary to focus on short-term effects on P_i_-signalling networks, and carefully monitor its tissue accumulation patterns to distinguish clearly between primary and secondary effects. This growth inhibition could also cause species loss in severely P-impoverished landscapes. At this time, it is also unclear whether different PHT transporters have different affinities for phosphite. As a first step, these kinetic properties would need to be determined and compared with the kinetics of P_i_ uptake in electrophysiological studies, as well as knock-out lines for individual transporters in *Arabidopsis* or other model plant species. As it is not clear whether any of the known PHT transporters also acts as a tranceptor that signals the availability of P_i_, similar to the nitrate-sensing properties of the AtNTR1;1 transporter (Gojon *et al.*, 2011), phosphite could directly interfere with this transceptor function, perturbing down-stream signalling pathways. Another possibility is that phosphite interferes with protein kinase/phosphatase regulatory units that control the phosphorylation status of PHT proteins, thereby regulating their incorporation into the plasma membrane (or their activity; Bayle *et al.*, 2011).

Phosphite competitively inhibits phosphate uptake by cluster roots in *Hakea sericea* (Sousa *et al.*, 2007). It also ­triggers cluster-root development alongside other P-starvation responses in P-sufficient white lupin (Gilbert *et al.*, 2000). Given the above-mentioned observations in model plant species, phosphite has the potential to suppress cluster-root development in P-limited Proteaceae and other cluster-root producing plant species. Thus, there could be direct implications for the phosphate-mining strategy of native Proteaceae species upon long-term exposure to phosphite. The exact time frame over which these changes might occur is unknown, and detailed monitoring of treated flora and plant communities is therefore recommended. Given that phosphite has been used for only 15 years, the tipping point may not yet have been reached.

**Figure 8:**
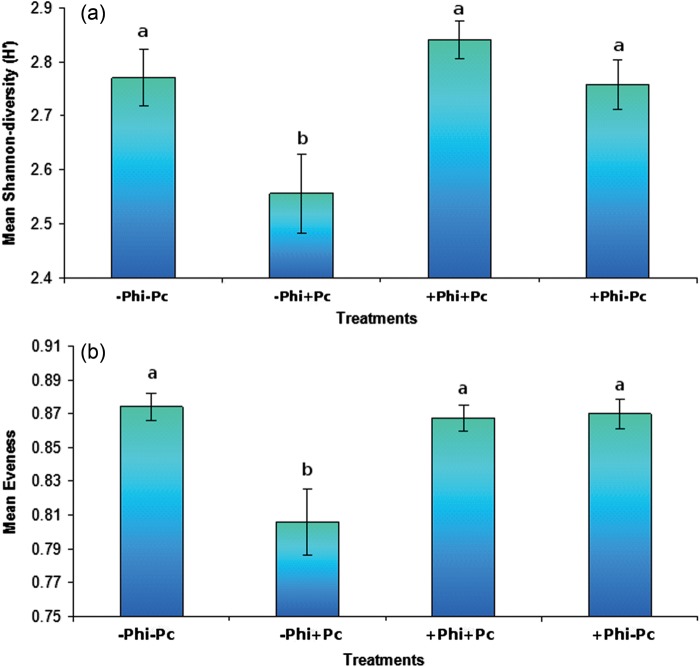
Mean Shannon-diversity (**a**) and evenness (**b**) for four ‘treatments’ (plus or minus *P. cinnamomi* and/or long-term phosphite spraying) at Gull Rock National Park, near Albany in Western Australia. The bars represent the SEM; *n* = 40. Mean Shannon-diversity index was significantly different between the four treatments (*F*(3,36) = 5.734, *P* = 0.003), as was the mean evenness (*F*(3, 36) = 8.111, *P* < 0.0001).

Even though phosphite applications benefit plant ­biodiversity in the shorter term by reducing dieback caused by *P. cinnamomi*, they may also lead to losses of P-sensitive or slow-growing plant species in the longer term. Very little information is available on the effects of long-term phosphite spraying in natural systems on species abundance and functioning. In a study in Gull Rock National Park, near Albany in Western Australia, a site exposed to phosphite application differs significantly from a control site without phosphite application in terms of species composition and diversity. The Shannon-diversity (Fig. 8a) index ranged from 0 (only one species is present, so no uncertainty to which species a randomly picked individual belongs) to 4.5 (where all the species are well represented, so there is high uncertainty; Hill, 1973; Keylock, 2005). The fewer the disturbances (i.e. *P. cinnamomi* infestation, phosphite spraying), the higher the index value. Evenness, on the other hand, represents how abundance is distributed among the different species within a community. An evenness index (Fig. 8b) closer to 0 indicates that most species are rare and only few are abundant (i.e. high dominance), while an index near to 1 means that all species have equal abundance (Hill, 1973; Keylock, 2005). The Shannon-diversity index suggests that the unsprayed and *P. cinnamomi*-infested site has the lowest species diversity when compared with the other three ‘treatments’, in accordance with other findings for the region (Bishop *et al.*, 2010). This means that it is difficult to predict which species a randomly picked individual belongs to compared with the other three treatments, because the plant species present are rare and sparsely distributed. The most distinct treatments, in terms of plant species composition and abundance, were the unsprayed and *P. cinnamomi*-infested plots and the phosphite-sprayed and *P. cinnamomi*-free plots, followed by the phosphite-sprayed and *P. cinnamomi*-infested plots and the phosphite-sprayed and *P. cinnamomi*-free plots. Assuming that the plots at very close distance were similar before phosphite applications and *P. cinnamomi* infestation, we are faced with the conclusion that both *P. cinnamomi* and phosphite have negatively impacted the biodiversity in Gull Rock National Park, which is representative of a significant component of south-western Australia's global biodiversity hotspot.

## Concluding remarks

Given that the greatest plant diversity in south-western Australia occurs on its most severely P-impoverished soils, P enrichment of natural habitats is a major threat to biodiversity of this globally significant biodiversity hotspot. We surmise that this threat is very similar for other P-impoverished environments, including the fynbos in South Africa (Cowling *et al.*, 1996; Richards *et al.*, 1997; Linder, 2005) and the cerrado in Brazil (Eiten, 1972; Ratter *et al.*, 1997; Lannes *et al.*, 2012). To conserve the biodiversity of such habitats requires maintenance of low soil fertility, in particular a low soil P status (Heddle and Specht, 1975; Adam, 2012). Minimizing the impact of run-off, P-containing fire suppressants, and pesticides, and maintaining a low fire frequency are therefore essential (Table 1). In old, climatically buffered, infertile landscapes, P is the key nutrient that provides a threat to plant biodiversity, rather than nitrogen, which tends to be a key limiting factor in younger landscapes (Hopper, 2009; Lambers *et al.*, 2010). Once these systems have been eutrophied, it is extremely difficult to restore their former glory (Standish *et al.*, 2008; Standish and Hobbs, 2010).

Whilst *P. cinnamomi* is a major threat to Australia's biodiversity, combating it by using phosphite cannot be a viable long-term solution, because P enrichment of severely P-impoverished soils replaces one threat by another (Table 1). In the short term, in the absence of any alternative, phosphite applications are essential. However, what is urgently required is a much better understanding of how phosphite functions, so that we can work towards alternatives for phosphite to achieve the same immunizing effects that phosphite induces in plants in response to oomycete pathogens. Once we know which metabolites are affected by phosphite, we can target the metabolic pathways with which phosphite interacts. Based on that knowledge, we can screen alternative, non-P-containing compounds for their ability to induce the same defence mechanisms, and thus attempt to induce the same anti-*Phytophthora* effects as phosphite.
